# Data Augmentation of a Corrosion Dataset for Defect Growth Prediction of Pipelines Using Conditional Tabular Generative Adversarial Networks

**DOI:** 10.3390/ma17051142

**Published:** 2024-03-01

**Authors:** Haonan Ma, Mengying Geng, Fan Wang, Wenyue Zheng, Yibo Ai, Weidong Zhang

**Affiliations:** 1 National Center for Materials Service Safety, University of Science and Technology Beijing, Beijing 100083, China; 2 Southern Marine Science and Engineering Guangdong Laboratory (Zhuhai), Zhuhai 519082, China

**Keywords:** corrosion depth, data augmentation, CTGAN, corroded pipeline, machine learning

## Abstract

Due to corrosion characteristics, there are data scarcity and uneven distribution in corrosion datasets, and collecting high-quality data is time-consuming and sometimes difficult. Therefore, this work introduces a novel data augmentation strategy using a conditional tabular generative adversarial network (CTGAN) for enhancing corrosion datasets of pipelines. Firstly, the corrosion dataset is subjected to data cleaning and variable correlation analysis. The CTGAN is then used to generate external environmental factors as input variables for corrosion growth prediction, and a hybrid model based on machine learning is employed to generate corrosion depth as an output variable. The fake data are merged with the original data to form the synthetic dataset. Finally, the proposed data augmentation strategy is verified by analyzing the synthetic dataset using different visualization methods and evaluation indicators. The results show that the synthetic and original datasets have similar distributions, and the data augmentation strategy can learn the distribution of real corrosion data and sample fake data that are highly similar to the real data. Predictive models trained on the synthetic dataset perform better than predictive models trained using only the original dataset. In comparative tests, the proposed strategy outperformed other data generation methods.

## 1. Introduction

Pipelines are the most economical and safest way of transporting oil and gas over long distances [1]. As pipelines undergo aging and coatings experience degradation, corrosion will occur on the outer surface of the buried pipeline [2,3]. Corrosion is a phenomenon characterized by the deterioration of metals resulting from electrochemical processes occurring on the metal surface exposed to its surrounding environment. The steady loss of pipe metal on the external surface of the pipeline will result in a reduction in its service life and compromise its structural integrity [4,5], which may cause significant losses to human safety and the economy and may also have a catastrophic impact on the environment (for example, through soil contamination, toxic spills, and explosions) [6].

The corrosion growth model can assess accurately the corrosion condition of buried pipelines, providing a scientific basis for pipeline integrity management [7,8]. In the framework of Industry 4.0, multiple systems that involve monitoring operations, managing risks, and maintaining and inspecting oil and gas pipelines are being extensively digitized [9]. This advancement facilitates rapid expansion and application of data-driven models for predicting corrosion damage in pipelines [10]. Data-driven models have the ability to learn linear or non-linear relationships between environmental factors and corrosion rate/depth from rich datasets. Ben Seghier et al. [2] used a range of artificial intelligence models to study the relationship between the maximum pitting depth and factors that contribute to pitting. Yazdi et al. [11] proposed a methodology for pipeline integrity management that utilizes a dynamic Bayesian network approach, taking into account the effects of microbial corrosion. Akhlaghi [6] explored the potential of deep learning models to predict the maximum pitting depth, with model training taking into account various characteristics of the soil as well as different types of coatings on the pipelines. However, data-driven models use historical data to train model parameters to predict future changes in corrosion depth. When constructing predictive models, it is imperative to give careful consideration not only to the advanced algorithm, but also to the dataset used to train and test the model used.

A reliable database should contain sufficient quantities of high-quality data, covering as much information as possible in the research. Due to long periods of corrosion, the high cost of detection and the privacy of the data, it is sometimes difficult to collect large amounts of real corrosion data comprising varying ages of pipelines and associated soil properties [12]. Therefore, most current models are established using corrosion data sourced from publicly available datasets. Existing datasets that contain information on soil parameters as well as the actual corrosion depths of long-life pipelines are the National Institute of Standards and Technology (NIST) dataset and Velázquez’s dataset [13]. The NIST dataset was obtained by field investigations conducted on several pipes that were buried in 128 locations across the United States. These studies spanned a period of up to 17 years and included soils that were representative of the various regions. The Velázquez dataset was obtained from 259 underground pipelines in operation in southern Mexico over the course of three years. The Velázquez’s dataset has extensive information on various kinds of coating and cathodic protection, in contrast to the NIST dataset, which only includes data from uncoated pipes without cathodic protection [14]. Thus, Velázquez’s dataset is regarded as a more accurate representation of actual pipeline corrosion and is highly favored by scholars. However, the quality and quantity of this dataset become a drawback that limits the performance of predictive models. Firstly, due to the spatial and temporal uncertainty of soil data, outliers will inevitably appear in the collected data. Outliers not only affect modeling accuracy but may also cause model overfitting. More importantly, the sample size of the dataset is too small and the distribution of coating styles is uneven, which may affect the generalizability and prediction accuracy of the prediction model. Unfortunately, none of these issues have received their due attention.

In cases where the size of the real dataset cannot be scaled up due to cost and data scarcity, the use of data augmentation techniques to generate fake data in place of the real data to improve model performance is an approach worth considering [15]. Common deep generative models include variational auto-encoders (VAEs), generative adversarial networks (GANs) and their variants. The GAN is a novel data augmentation approach that has been recently developed to increase the sample variety and sample size based on real data and has been widely applied in the fields of image and classification. It is an improvement on VAE, and the accuracy of generated samples is better than in other synthetic data oversampling methods [16]. Douzas and Bacao [17] used the conditional version of generative adversarial networks (CGANs) to approximate the real data distribution and fake data for the minority class of various imbalanced datasets. Tang et al. [18] used CGANs and CTGANs to generate more formation characteristics data for shale reservoirs through a small number of samples. He and Zhou [19] used tabular generative adversarial networks to generate synthetic full-scale blasting test data for corroded pipelines. Woldesellasse and Tesfamariam [20] used a CGAN to handle the class imbalance in soil data by generating synthetic samples. Habibi et al. [21] used a CTGAN and machine learning for imbalanced tabular data modeling to improve IoT botnet attack detection. However, data augmentation research for regression has received less attention than for classification, especially for tabular data that contain both continuous data and discrete data with uneven distributions. Datasets for regression contain both input and output variables. When a GAN is used directly to generate corrosion data, it does not distinguish between input and output variables, but treats each equally [22]. This will result in a situation where the generated output variables do not match up with the generated input variables, even when the generated input variables have a similar distribution and correlation with the real input variables. As a result, the overall quality of the generated dataset is reduced, and models built on the synthetic dataset comprising these generated data may exhibit low prediction accuracy.

The primary objective of this work is to explore the use of data augmentation to generate corrosion data, thereby furnishing an accurate and robust dataset for predicting the corrosion depth growth in corroded pipelines. The research endeavors to address the following challenges:

1. Outliers’ detection: deleting anomalous data points in the original corrosion dataset.
2. Tabular data handling: The corrosion dataset consists of data with a variety of structures and has different distributions for continuous variables and uneven distributions for discrete variables.
3. Oversampling: Ensuring that the model can capture the real data distribution and generate new samples following the same distribution. If the real data are randomly sampled during the training process, the rows with the smallest number of categories will not be fully represented, so the GAN may not be trained correctly. If the real data are oversampled, the GAN will learn the oversampled distribution instead of the real data distribution.
4. Regression challenges: It is imperative that the relationship between environmental factors and corrosion depth remain unchanged in the generated data.

Therefore, a novel data augmentation strategy is proposed in this paper. The data generation in this strategy consists of two parts: the generation of input variables (environmental factors) and output variables (corrosion depth) for corrosion growth prediction. For the outliers in the corrosion dataset, multiple detection methods are used for data cleaning. For tabular data and oversampling, CTGAN, which introduces mode-specific normalization and training by sampling, is used to learn real data to generate new environmental factors. For the regression challenge, a corrosion growth model based on machine learning algorithms is applied to generate corrosion depths corresponding to the new environmental factors. The fake data are merged with the original data to form the synthetic dataset. Finally, the effectiveness of the proposed strategy is verified by analyzing the distribution and credibility of the synthetic dataset using various visualization methods and evaluation metrics. This technique can provide data support for future corrosion growth modeling, and this method can also be applied to data generation for regression problems in other fields.

The rest of this paper is structured as follows. In Section 2, the buried pipeline corrosion dataset is introduced, and data cleaning and correlation analysis are performed on this dataset. Section 3 proposes the data augmentation strategy and introduces the theory of key algorithms. Section 4 verifies the credibility of the synthetic data and the advancement of the strategy. In Section 5, the main work of this research is summarized.

## 2. The Buried Pipeline Corrosion Dataset

The corrosion dataset utilized in this study comprises 241 samples derived from Velázquez’s dataset. The maximum pitting depth (*d*_max_), soil properties, pipe age (*t*), coating type (*ct*), and pipe-to-soil potential (*pp*) were recorded for each sample. In this dataset, any metal loss resulting from corrosion with a diameter equal to or less than twice the wall thickness of the pipeline is denoted the maximum pitting depth. The soil properties include pH, soil resistivity (*re*), water content (*wc*), redox potential (*rp*), chloride content (*cc*), sulfate content (*sc*), bicarbonate content (*bc*), bulk density (*bd*), and soil texture (*class*). The variables *ct* and *class* in the dataset are discrete. The coating types include non-coated (NC), asphalt–enamel-coated (AEC), wrap tape-coated (WTC), coal tar-coated (CTC), and fusion-bonded epoxy (FBE). The soil texture is classified according to U.S. Department of Agriculture standards into clay (C), sandy clay loam (SCL), or clay loam (CL) [23]. Table 1 lists the statistics of buried pipeline corrosion data. In continuous variables, Mean, Min, Max and std. represent the mean, minimum, maximum and standard deviation of the variable, respectively. It is evident that the majority of variables do not conform to a normal distribution. In discrete variables, min and max represent the smallest and largest number of categories in the variable. It is evident that there exists a substantial disparity in the numerical distribution of *ct* categories, with certain categories exhibiting a notably low count. Among them, the number of NC is 31, the number of AEC is 6, the number of WTC is 95, the number of CTC is 101, and the number of FBE is 8.

### 2.1. Data Cleaning

In statistics, the outlier is an observation that differs from other well-structured data. Common outlier detection methods include statistics-based methods, distance-based methods, clustering-based methods, and model-based methods [24]. To mitigate the risk of over-reliance on a single method and excessive elimination of data, this paper identifies one technique from each of these four categories based on the distribution and characteristics of the variables. A data point is an outlier if it is determined to be an outlier by three of these four techniques.

In statistics-based methods, the interquartile range is used to measure statistical dispersion and data variability by dividing the dataset into quartiles [25].

In distance-based methods, the Mahalanobis distance is used to determine whether a data point is an outlier by calculating the distance between the data point and other data points.

In the clustering-based method, DBSCAN is used to cluster the data points into different clusters and then determine whether the data points are outliers or not by judging whether they belong to a certain cluster [26].

In the model-based method, quantile regression is used to describe the distribution of data at different quantiles by fitting regression lines at different quantiles, thereby identifying data points that deviate greatly from the normal situation [27].

**Figure 1:**
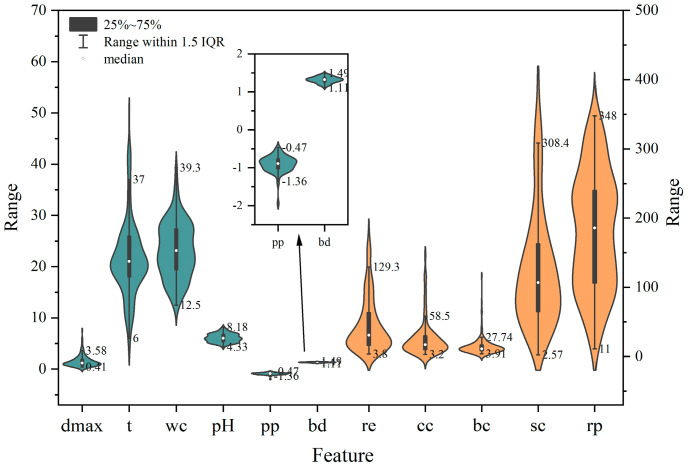
The violin plot of the modified dataset.

Taking into account the physical meaning of the variables, this paper deletes a total of 43 outliers. In order to illustrate the statistical properties of the modified dataset and its probability density distribution, that is, the median and quartiles, upper limit and lower limit, etc., the violin plot is drawn. The violin plot is a graphical technique used to represent continuous data and can be considered a combination of box plots and kernel density plots. As depicted in Figure 1, the data distribution after outlier detection is more concentrated.

### 2.2. Relational Analysis

The Spearman correlation coefficient is used to assess the monotonic relationship between variables when analyzing the correlation between variables in soil properties. It is a statistical measure that is applicable in situations when the relationship between variables is non-linear or when the variables do not adhere to a normal distribution [28]. The Spearman correlation coefficient does not assume that the data come from a specific distribution, which makes it more flexible in terms of the form and distribution of the data.

(1) $$\rho =\frac{\sum \left(R(x_{i})-\bar{R(x)}\right)\left(R(y_{i})-\bar{R(y)}\right)}{\sqrt{\sum {\left(R(x_{i})-\bar{R(x)}\right)}^{2}\sum {\left(R(y_{i})-\bar{R(y)}\right)}^{2}}}$$

where $R(x_{i})$ and $R(y_{i})$ are the levels of the *i*-th observation of *x* and *y*. $\bar{R(x)}$ and $\bar{R(y)}$ are their average values.

**Figure 2:**
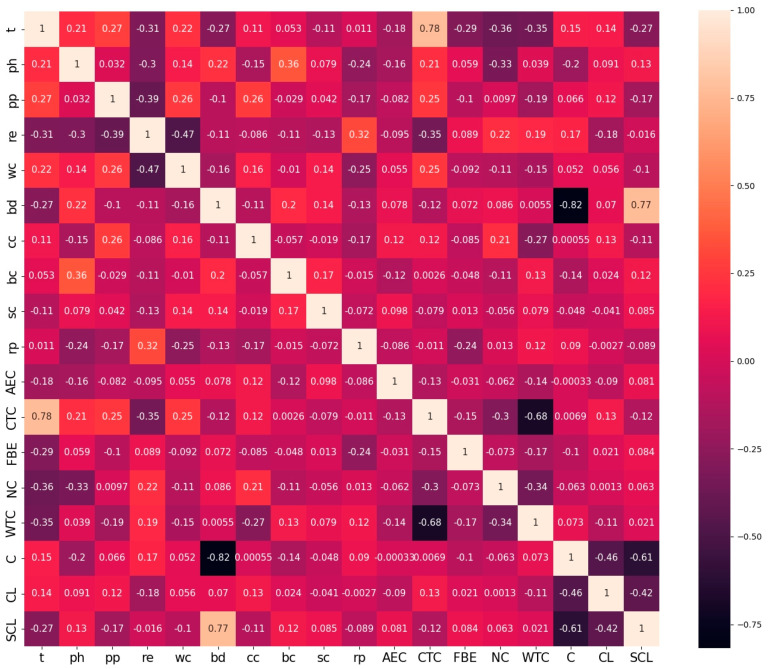
The Spearman correlation coefficient between variables in soil properties.

The Spearman correlation coefficients between variables in soil properties are depicted in Figure 2. The correlation coefficient ranges from −1 to 1. The strength of the correlation between the two variables increases as the value approaches 1 or −1. Positive values signify a positive correlation, whereas negative values signify a negative correlation. In continuous variables, the relationship between soil resistivity and water content exhibits a strong negative association. The remaining variables exhibit relatively weak correlations.

## 3. Methodology

### 3.1. The Proposed Data Augmentation Strategy

The data augmentation strategy proposed in this paper is divided into two modules. The first module that of data generation, which combines machine learning and deep generative models. Its main task is to generate data on environmental factors as well as corrosion depth. The focus is to ensure that the correlation and joint probability density between the input variables of the fake dataset are the same as those of the original dataset, while ensuring that the output variables still correspond to the input variables. The second module is data verification. The synthetic dataset is analyzed using a variety of evaluation indicators.

**Figure 3:**
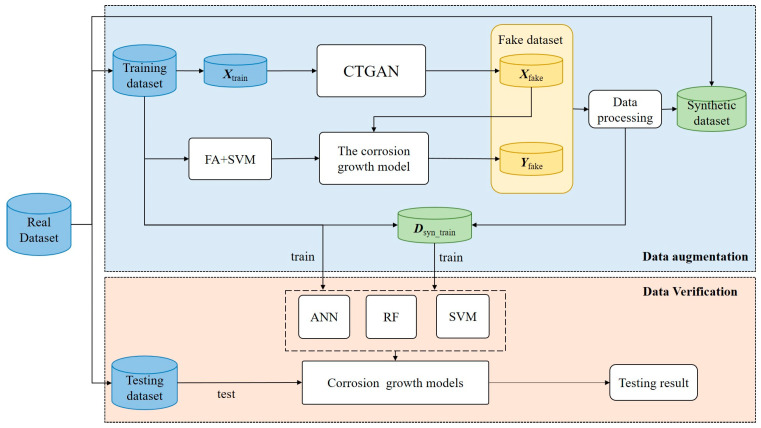
The proposed data augmentation strategy.

The details of this strategy are shown in Figure 3. The corrosion dataset after data cleaning is divided into a training set and a testing set [29]. The training set is used to generate fake data and the testing set is used to verify the credibility of the synthetic dataset. Since the corrosion dataset is tabular data and is not unevenly distributed, CTGAN is introduced to learn the distribution and correlation of the input variables ***X***_train_ in the real data to generate fake data ***X***_fake_ similar to real data. In order to generate accurate output variables, this paper employs a hybrid method integrating a support vector machine with a firefly algorithm (FA) to establish a corrosion growth model, which has been proven by El Amine Ben Seghier et al. [30] to be able to obtain corrosion depth with higher prediction accuracy than other methods. The ***X***_fake_ is input to the corrosion growth model to obtain the corresponding ***Y***_fake_. ***X***_fake_ and ***Y***_fake_ together form the fake dataset. Data from the fake dataset that do not conform to the physical meaning of the variables are eliminated, such as the data with pH or *t* less than 0. The fake dataset is merged with the original dataset to form a synthetic dataset. To verify the credibility of the synthetic dataset, the dataset ***D***_syn_train_ integrating the fake dataset with the training set is used to establish corrosion growth models based on three different machine learning algorithms, and the predictive performance of these models on the testing set is compared with that of a corrosion growth model built using only the training set.

### 3.2. Deep Generative Models

#### 3.2.1. Generative Adversarial Networks

**Figure 4:**
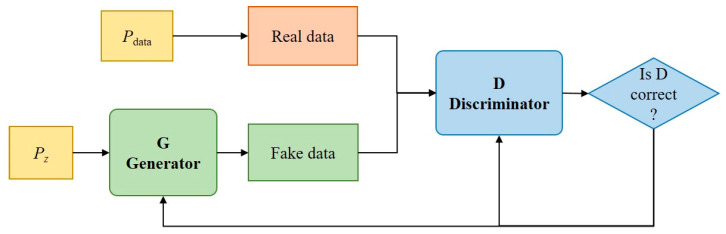
The structure of GAN.

GAN is a framework designed to train generative models using adversarial techniques [31]. It jointly optimizes two models, namely the generator **G** and the discriminator **D**. The structure can be observed in Figure 4. Both models are independent neural networks. The generator takes random noise as input and captures the distribution of the training dataset. Its objective is to generate fake data that are indistinguishable from real data to fool the discriminator. Both the fake data and real data will then be simultaneously inputted into the discriminator. The objective of the discriminator is to differentiate between fake data and real data, with the discerned outcomes subsequently being sent as feedback to the generator. Throughout the training of GAN, the **G** and **D** continuously learn from each other through adversarial interaction. Once the generator has successfully learned knowledge of the distribution of real data and generates fake data that closely resemble the real ones, the discriminator will no longer be able to properly discern the legitimacy of the input samples, indicating that the training has been completed.

The optimization process of GAN is essentially a game process, wherein the objective is to identify the extremum and achieve equilibrium. The distribution of noise *z* is denoted as $P_{z}(z)$, while the distribution of real data is denoted as $P_{data}(x)$. D(x) represents the probability that the sample comes from real data, taking on values between 0 and 1. G(z) represents the data generated by **G** through the learning process [32]. To attain equilibrium between the **G** and **D**, the objective function of GAN is formulated as follows [31]:

(2) $$\underset{G}{\min}\;\underset{D}{\max}V\left(D,G\right)=E_{x\sim p_{data}(x)}[\log D(x)]+E_{x\sim p_{z}(z)}[\log(1-D(G(z)))]$$

where the first item indicates the discriminator probability of the training data, and the second item indicates the discriminator prediction of the fake data.

#### 3.2.2. Conditional Tabular Generative Adversarial Networks

The corrosion dataset comprises 11 columns of continuous variables and 2 columns of discrete variables. Every column can be considered a random variable following an unidentified distribution, whereas each row can be seen as a specific instance of the joint probability distribution of each variable. Hence, the corrosion dataset presents various distinctive characteristics that pose a challenge to the GAN model. These include the presence of mixed data types, non-Gaussian and multimodal distributions, and highly imbalanced categorical columns. 

Therefore, this paper introduces CTGAN to address these problems, which carries out the mode-specific normalization and designs a conditional generator and training-by-sampling [33].

The variational Gaussian mixture model is employed to estimate the number of patterns and fit a Gaussian mixture for every continuous column $C_{i}$.

(3) $${\mathbb{P}}_{C_{i}}\left(c_{i,j}\right)=\sum_{k=1}^{m}\mu_{k}N\left(c_{i,j};\eta_{k},\phi_{k}\right)$$

where $c_{i,j}$ is the value of the *i*-th column and *j*-th row. $\mu_{k}$, $\eta_{k}$ and $\phi_{k}$ are the weights, means, and standard deviations of *k*th modes, respectively.

Therefore, the probability of each value $c_{i,j}$ coming from each mode can be calculated by the probability densities $\rho_{k}=\mu_{k}N\left(c_{i,j};\eta_{k},\phi_{k}\right)$ of each pattern. $c_{i,j}$ is represented as a one-hot encoded vector $\beta_{i,j}$ specifying the pattern and a scalar $\alpha_{i,j}$ referring to the specific value in the modes.

(4) $$\alpha_{i,j}=\frac{c_{i,j}-\eta_{k}}{4\cdot \phi_{k}}$$

For the imbalance problem in categorical columns, the vector ***cond*** is introduced to indicate the condition. There are two discrete columns in the corrosion dataset (the coating and soil categories), namely $D_{1}=\left\{1,2,3,4,5\right\}$ and $D_{2}=\left\{1,2,3\right\}$. The discrete columns ***D*_1_** and ***D*****_2_** are represented as one-hot vectors ***d*_1_** and ***d*_2_**. The mask vector ***m*_i_** is utilized to denote the associated one-hot vector ***d*_i_**. For example, if specifying the third coating as a condition, we have $m_{1}=\left[0,0,1,0,0\right]$, $m_{2}=\left[0,0,0\right]$, and $cond=\left[0,0,1,0,0,0,0,0\right]$. In addition, the cross-entropy between ***m*_i_** and ***d*_i_** is added to penalize its loss to force the conditional generator to produce ***d*_i_** = ***m*_i_**. 

The generator G and discriminator D of CTGAN use the fully connected network to capture all possible relationships between columns. There are two fully connected hidden layers in the network structure. Moreover, the generator G uses the batch normalization and the Relu activation function [33].

### 3.3. Machine Learning

In order to mitigate the risks associated with depending on a single algorithm style, three types of machine learning methods were chosen to validate the synthetic dataset. These algorithms include the artificial neural network (ANN), which is based on a neural network; random forest (RF), which is based on decision trees; and the support vector machine (SVM).

(1) Artificial neural networks

Artificial neural networks comprise several neuron nodes, which consist of the input layer, hidden layer, and output layer. Neurons in the hidden layer establish connections between the input and output layers using specific nonlinear functions [34]. Neural networks can make precise predictions by being trained on a set of sample data.

(5) $$\begin{array}{l} Y=b+\sum_{j=1}^{n}w_{j}\phi_{j}\\ \phi_{j}=\frac{1}{1+\exp\left[-\left(b_{j}+\sum_{i=1}^{n}w_{ji}x_{i}\right)\right]} \end{array}$$

where b and $b_{j}$, respectively, represent the bias for the output and the *j*-th hidden nodes. $w_{j}$ and $w_{ji}$, respectively, denote the weights for the output and hidden nodes.

(2) Random forest

The fundamental principle of RF is to construct many base models and combine their predictions in order to achieve precise outcomes. RF employs bagging and random attribute selection techniques for constructing models [35]. The decision trees are constructed by obtaining samples and will cease to branch further if their mean squared error reaches the optimal level. The predictions from each decision tree are tallied, and the outcomes are collectively voted upon to generate the ultimate conclusion.

(3) Support vector machine

The concept of SVM is to partition a dataset to determine the hyperplane with the largest geometric separation. The hyperplane is defined using Equation (6).

(6) $$f\left(x\right)=\langle \omega ,x\rangle +b$$

where ω,x represent the regression coefficient vector and bias. The learning strategy of SVM is to maximize the interval, which can be formalized as a problem of solving convex quadratic programming.

### 3.4. Evaluation Indicators

There is no unified evaluation indicator for adversarial generation networks. In this work, a combination of statistical analysis and machine learning methods is applied to evaluate the performance of the proposed strategies.

This paper performs statistical analysis on the synthetic dataset and explores correlations between variables in the dataset. The Kolmogorov–Smirnov (KS) test is used to measure whether real data $F_{1}(x)$ and fake data $F_{2}(x)$ come from the same distribution [36].

(7) $$D=\sup_{x}\left|\left.F_{1}(x)-F_{2}(x)\right|\right.$$

The credibility of synthetic datasets is evaluated by machine learning models. Therefore, mean absolute error (MAE), root mean square error (RMSE), mean absolute percentage error (MAPE) and the correlation coefficient (*R*^2^) are selected as evaluation indicators. MAE and RMSE represent the deviation between the predicted value and field data. MAPE refers to the deviation between the predicted value and field data as a percentage of field data. *R*^2^ shows the fit between the predicted value and the field data.

(8) $$\mathrm{RMSE}=\sqrt{\frac{1}{n}\sum_{i=1}^{n}{\left(y_{i}-{\hat{y}}_{i}\right)}^{2}}$$

(9) $$\mathrm{MAE}=\frac{1}{n}\sum_{i=1}^{n}\left|y_{i}-{\hat{y}}_{i}\right|$$

(10) $$\mathrm{MAPE}=\frac{100\%}{n}\sum_{i=1}^{n}\left|\frac{y_{i}-{\hat{y}}_{i}}{y_{i}}\right|$$

(11) $$R^{2}=1-\frac{\sum_{i=1}^{n}{\left(y_{i}-{\hat{y}}_{i}\right)}^{2}}{\sum_{i=1}^{n}{\left(y_{i}-\bar{y}\right)}^{2}}$$

In Equations (8)–(11), $y_{i}$, ${\hat{y}}_{i}$, $\bar{y}$ are the field data, predicted data, and average values of predicted data; *n* is the number of the dataset.

## 4. Results and Discussions

The corrosion dataset after data cleaning contains a total of 197 data points; 70% of the data are used for the training set ***D***_train_, whereas 30% of the data are used for the testing set ***D***_test_. The ***D***_train_ and ***D***_test_ sets consist of 137 and 60 data points, respectively. The data analysis and model training are performed using the algorithm packages contained in Python 3.8.

### 4.1. The Evaluation of the Synthetic Dataset

Before using data augmentation to generate the fake data, the hyperparameters of the algorithm in strategy need to be tuned. The main hyperparameters that affect the performance of CTGAN are the learning rate of the generator and the discriminator, epoch, and batch size. This paper uses the Bayesian optimization algorithm to optimize the hyperparameters. Bayesian optimization is a method that determines the best combination of hyperparameters by using various surrogate functions to fit the relationship between the hyperparameters and evaluation [37]. In this paper, the tree-structured Parzen estimator is chosen as the surrogate function, and the sum of the KS statistics of fake data and real data for each variable is used for the evaluation. The smaller the sum of the KS statistic, the closer the distribution of fake data and real data is. The total number of epochs is 1750 and the batch size is 150. The learning rates of generator G and discriminator D are 0.00037 and 0.000223, respectively. The main hyperparameters that affect the performance of SVM are regularization parameter C and epsilon, which are tuned by the FA algorithm. The regularization parameter C is 1.198 and epsilon is 0.1691.

The input variables ***X***_train_ in the training set are fed into CTGAN, and AEC and FEB in *ct* are selected as conditional vector ***cond*** to generate 200 new data points each. After excluding negative values in other variables from the data except *pp*, a total of 378 ***X***_fake_ samples were obtained. The training set ***D***_train_ is used to train SVM to establish the corrosion growth model. ***X***_fake_ is used to input the corrosion growth model to obtain ***Y***_fake_, and the fake dataset ***D***_fake_ is finally obtained. The original dataset and the fake dataset are merged into a synthetic dataset ***D***_syn_, with a total of 575 data points. Its statistics are shown in Table 2.

**Figure 5:**
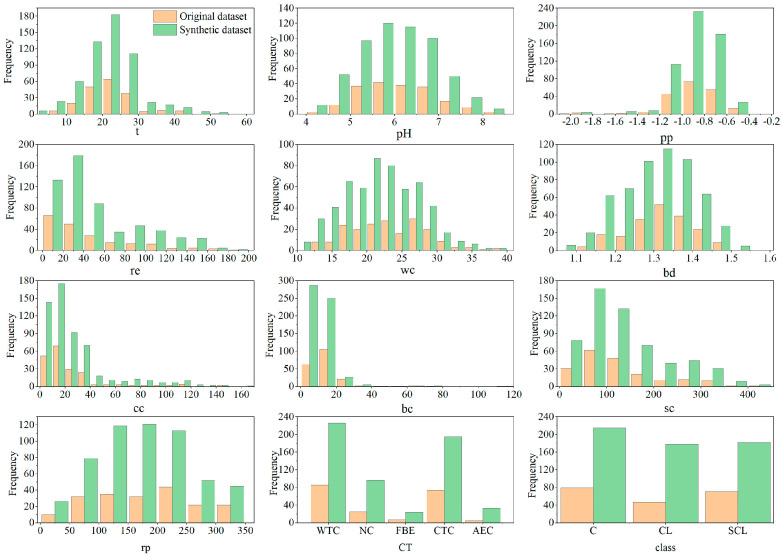
Histogram of the frequency distribution of the variable.

In order to evaluate whether the proposed data augmentation strategy can truly learn and generate real corrosion data, different visualization methods were used to analyze the synthetic dataset. First, the distribution of each input variable between the original dataset and the synthetic dataset is compared visually, as shown in Figure 5. The original dataset is represented in orange and the synthetic dataset is represented in green. For continuous variables, the original dataset and the synthetic dataset have similar distributions. For discrete variables *ct*, the data on FBE and AEC have been supplemented. Therefore, the statistical properties of the synthetic dataset closely match those of the original dataset.

**Figure 6:**
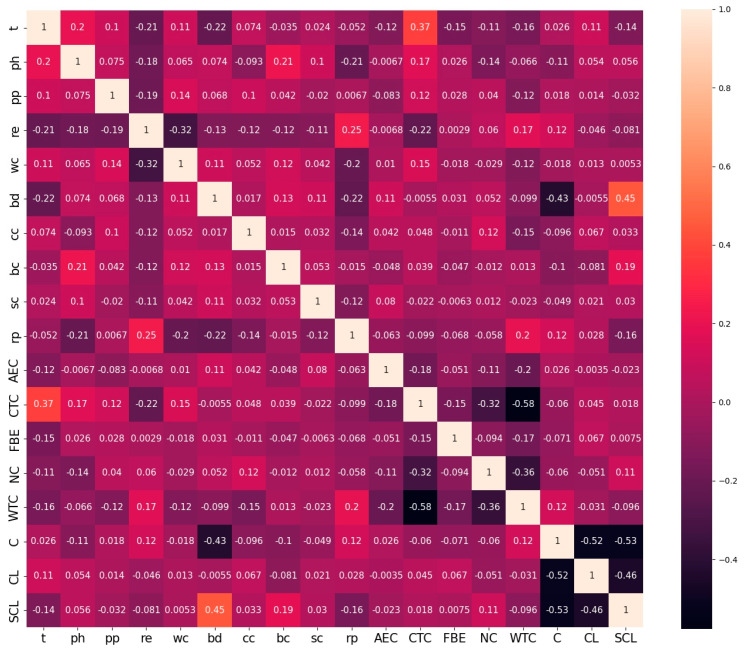
Heat map of the Spearman correlation coefficient of the synthetic dataset.

Then, Spearman correlation analysis was performed on the synthetic dataset, as depicted in Figure 6. The result shows that compared to the correlation matrix of the original dataset, the synthetic dataset is able to keep the relationship between the variables of the original dataset. This indicates that the proposed strategy is able to generate data with a similar structure to the real data.

**Figure 7:**
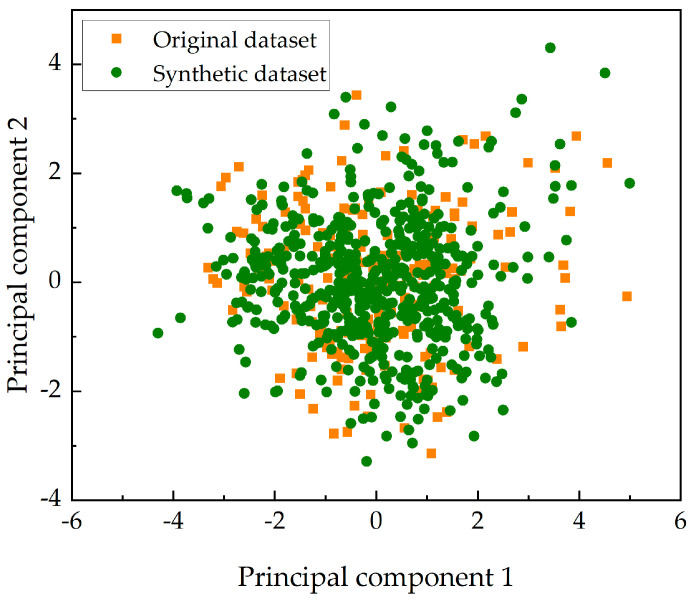
The principal components of the original dataset and synthetic dataset.

There are 10 input variables in the synthetic dataset. For multidimensional data, principal component analysis can be used to extract principal components from the dataset to reduce the data dimension, which is a method of orthogonally transforming a set of variables into a set of linearly uncorrelated orthogonal basis principal components. Two principal components are extracted to represent multidimensional data from the original dataset and the synthetic dataset. The data are displayed in two-dimensional space, as depicted in Figure 7. It can be seen that the synthetic dataset has a similar distribution to the original dataset. Therefore, this strategy can successfully capture the distribution of real corrosion data and sample fake data that are highly similar to the real data.

### 4.2. The Credibility of the Synthetic Dataset

Since corrosion data augmentation involves regression problems, it is necessary to ensure that the generated input variables and output variables still have the same correlation as in the original dataset. Therefore, this paper compares the predictive performance of models trained using synthetic data with that of models trained using real data and uses different types of machine learning for robustness training. First, ***D***_fake_ and ***D***_train_ are merged into the training set ***D***_syn_train_ of the synthetic dataset. Then, ***D***_train_ and ***D***_syn_train_ are used to train the three machine learning algorithms (ANN, RF, and SVM) to establish the corrosion growth models. The hyperparameters of three machine learning algorithms use default values. Finally, ***D***_test_ is used to test the predictive performance of these corrosion growth models to assess the credibility of the synthetic data. In addition, in order to further analyze the prediction performance of the model under each coating type, this paper classifies ***D***_test_ according to coating styles.

**Figure 8:**
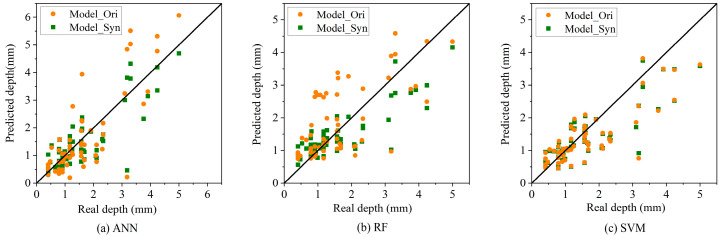
Comparison of predicted and real corrosion depth using Model_Syn and Model_Ori.

Table 3 depicts the prediction performances of the models Model_Ori trained by using ***D***_train_ and the prediction performances of models Model_Syn trained by using ***D***_syn_train_ under different coating styles. The smaller the values of MSE, MAE, and MAPE, the higher the prediction accuracy. The closer the *R*^2^ value is to 1, the better the fitting performance. For each *ct*, the prediction performances of Model_Syn are better than those of Model_Ori. Since the number of FBE and AEC in the original dataset is very small, the numbers of them in ***D***_test_ are only 2 and 1, respectively. Therefore, the prediction performances of those models under these two coatings are similar and the correlation coefficient cannot be calculated under AEC. For all coatings, the prediction performances of Model_Syn are significantly improved compared to the prediction performances of Model_Ori. Figure 8 further depicts the comparison of predicted and real corrosion depth between Model_Syn and Model_Ori. The diagonal line indicates that the predicted depth is equal to the real depth. It can be observed that the points of Model_Syn are closer to the diagonal line. Therefore, the synthetic dataset derived from the data augmentation strategy proposed in this paper shows high superiority in establishing the corrosion growth model, and the model shows certain improvements in prediction performance for each coating. The synthetic dataset can be used as a substitute for real data for the purpose of corrosion growth prediction.

### 4.3. Comparison with Other Data Generation Methods

The corrosion depth can also be generated through methods such as deep generative models or empirical formulas. Therefore, in order to analyze the difference between the synthetic dataset obtained by the method proposed in this paper and the synthetic dataset obtained by other methods, this paper compares the prediction performance of the corrosion growth model trained on the synthetic data obtained by different methods. To improve the quality of generated output variables, this experiment made some adjustments based on the proposed strategy. For example, when optimizing the hyperparameters of CTGAN, the evaluation is selected as the RMSE of the predicted depth and the real depth, and five-fold cross-validation is used to adjust the hyperparameters of CTGAN. The total number of epochs is 1300 and the batch size is 100. The learning rate of generator G is 0.000269, and the learning rate of discriminator D is 0.000227.

(1) The generation of corrosion depth through the deep generative model

**Figure 9:**
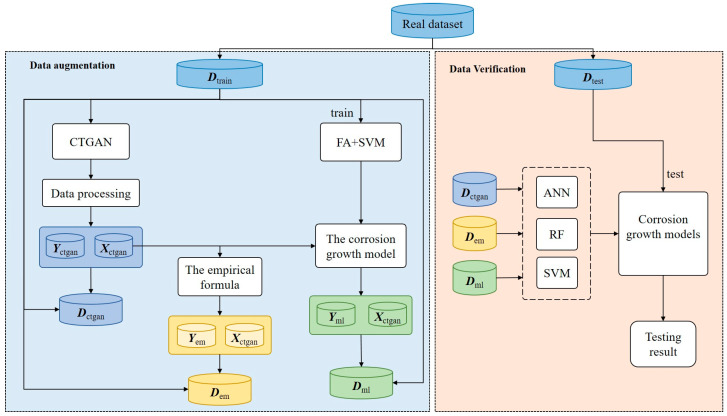
The framework for comparing with other data generation methods.

***D***_train_ is inputted to CTGAN, and AEC and FBE in *ct* are selected as the conditional vector ***cond*** to generate 300 new samples each. Negative values in other variables except *pp* are eliminated, as depicted in Figure 9. Since CTGAN is prone to producing some outliers, data whose absolute value of the difference between fake values and real values is greater than 0.5 times the real value will be deleted. Among them, the real value is replaced by the value obtained from the empirical formula. This measure also improves the quality of data generated by deep generative models. Finally, the data ***X***_ctgan_ and ***Y***_ctgan_ are obtained through CTGAN and merged with ***D***_train_ to form the synthetic dataset ***D***_ctgan_.

(2) The generation of corrosion depth through an empirical formula

The power function corrosion growth model is one of the most successful empirical models. Caleyo et al. [38] used multiple nonlinear regression analysis to establish a corrosion growth model based on the power function, which is widely used in current research.

(12) $$d_{\max}=\left(k_{0}+\sum_{i=1}^{n}k_{i}x_{i}\right){\left(t-t_{0}\right)}^{n_{0}+\sum_{j=1}^{m}n_{j}x_{j}}$$

where $t_{0}$ is the pit initial time, $x_{i}$ represents environmental factors, and $k_{i}$ and $n_{j}$ are the regression coefficient corresponding to each variable.

For different soil categories, $k_{i}$ and $n_{j}$ select corresponding values, as shown in Table 4. In Equation (12), the influence of coating is modeled through an independent variable whose value is assigned by means of the scoring model, namely FBE = 0.3, AEC = 0.9, WTC = 0.8, NC = 1, CTC = 0.7. ***X***_ctgan_ is inputted into Equation (12) to obtain ***Y***_em_. They are merged with ***D***_train_ to form the synthetic dataset ***D***_em_.

(3) The generation of corrosion depth through the proposed strategy

***X***_ctgan_ is input into the corrosion growth model based on SVM to obtain ***Y***_ml_. They are merged with ***D***_train_ to form the synthetic dataset ***D***_ml_.

**Figure 10:**
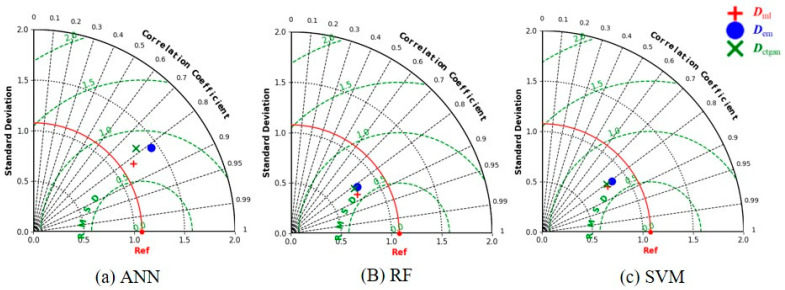
Taylor diagrams for comparison of models.

Three machine learning algorithms (ANN, RF and SVM) are trained using ***D***_ctgan_, ***D***_em_ and ***D***_ml_, respectively, to obtain the corrosion growth models, and ***D***_test_ is used to verify the prediction performance of these models. Table 5 shows that the predictive performance of the three machine learning algorithms trained on synthetic data obtained by the proposed strategy is better than that of models trained on the synthetic data generated by deep generative models and empirical formulas. Taylor diagrams can show the standard deviation, root mean square and correlation coefficient of the predicted values. This correlation coefficient refers to the Pearson correlation coefficient. The higher the correlation coefficient, the better the model’s prediction performance. Figure 10 shows Taylor diagrams of three machine learning models trained using three datasets. No matter what machine learning algorithm is used, the model built using ***D***_ml_ has smaller errors and a higher correlation. Therefore, it can be said that the strategy proposed in this paper is better than the empirical formula and single-CTGAN method.

## 5. Conclusions

In order to achieve an accurate prediction of corrosion depth growth, the data shortage problem in the establishment of data-driven models needs to be solved. Due to the non-Gaussian distribution of continuous variables and uneven distributions of discrete variables in the corrosion dataset, this paper proposes a data augmentation strategy using CTGAN to develop an accurate and robust dataset. Before data generation, data cleaning is performed using various outlier detection methods, and variables are analyzed using Spearman correlation coefficients. The input variables for predicting corrosion growth are generated using CTGAN, while the output variables corresponding to the input variables are generated using a corrosion growth model based on a hybrid method. The fake data and real data are merged into a synthetic dataset. To verify the effectiveness of the proposed strategy, the synthetic dataset is analyzed and tested. The conclusion is as follows:

(1) The proposed strategy can capture the real corrosion data and generate fake data that are the same as the real data. The variables in the synthetic dataset have similar distributions and Spearman correlation coefficients as the real dataset, and the two principal components obtained by the principal component analysis of the two datasets are similar.
(2) The corrosion growth models established by using the synthetic dataset have better predictive performance than the models established by using the real dataset for any coating type. Therefore, the synthetic dataset can be used as a supplement to the real data for corrosion growth prediction.
(3) The superiority of the proposed strategy is demonstrated by comparing it with existing deep generative models and empirical formulation methods. The results of this comparison show that the corrosion growth models established by using the synthetic dataset obtained by the proposed method have better prediction performance than those obtained via other methods.

## Figures and Tables

**Table 1:** The statistics of buried pipeline corrosion dataset.

Para.	Unit	Mean	Min	Max	Std.
*d*_max_	[mm]	1.925	0.41	10.41	1.842
*t*	[years]	22.77	5	50	9.02
*pH*		6.124	4.14	9.88	0.921
*wc*	[%]	23.73	8.8	57.6	6.16
*re*	[Ω·m]	49.35	1.9	399.5	53.99
*cc*	[ppm]	41.28	3.2	351.0	55.66
*sc*	[ppm]	152.38	1.0	1370.2	158.87
*bc*	[ppm]	18.43	1.0	156.2	20.82
*bd*	[g/mL]	1.304	1.11	1.55	0.086
*pp*	[mV]	−0.879	−1.97	−0.42	0.234
*rp*	[mV]	170.20	2.1	348.0	86.17
*ct*		48.2	6	101	
class		80.3	59	107	

**Table 2:** The statistics of the synthetic dataset.

Para.	Mean	Min	Max	Std.
*d*_max_	1.448	0.407	7.59	0.784
*t*	21.46	2	51	7.83
pH	6.044	4.03	8.18	0.850
*wc*	21.95	8.43	39.3	5.42
*re*	51.81	2.65	208.7	41.66
*cc*	27.72	0.34	164.1	28.09
*sc*	138.24	1.48	457.7	91.38
*bc*	18.43	0.29	117.2	8.96
*bd*	1.306	1.04	1.51	0.093
*pp*	−0.892	−2.65	−0.47	0.225
*rp*	181.16	0.07	423.6	85.24
*ct*	115	24	226	
*class*	191	178	215	

**Table 3:** Prediction performances of models built with original and synthetic datasets under different coating types.

ct			MAE	RMSE	MAPE	R^2^
WTC	Model_Ori	ANN	0.5169	0.7934	0.4000	−0.3026
		RF	0.5842	0.8080	0.5970	−0.3511
		SVM	0.3906	0.6119	0.3271	0.2251
	Model_Syn	ANN	0.3997	0.6519	0.3299	0.1206
		RF	0.3974	0.5603	0.4081	0.3504
		SVM	0.3853	0.5957	0.3271	0.2658
NC	Model_Ori	ANN	0.3663	0.4587	0.1518	0.8069
		RF	0.7487	0.8960	0.4516	0.2631
		SVM	0.6308	0.7768	0.2908	0.4462
	Model_Syn	ANN	0.5602	0.6971	0.2336	0.5539
		RF	0.5408	0.6302	0.2367	0.6354
		SVM	0.6269	0.7707	0.2889	0.4547
CTC	Model_Ori	ANN	0.8785	1.0958	0.4995	0.2603
		RF	0.8463	1.0372	0.5398	0.3373
		SVM	0.6057	0.7530	0.3222	0.6508
	Model_Syn	ANN	0.5238	0.6342	0.3504	0.7522
		RF	0.6062	0.7881	0.3179	0.6174
		SVM	0.6098	0.7631	0.3156	0.6413
FBE	Model_Ori	ANN	0.4879	0.4885	0.4291	−1.1911
		RF	0.2419	0.2820	0.1765	0.2696
		SVM	0.1170	0.1368	0.0852	0.8282
	Model_Syn	ANN	0.2148	0.2367	0.2094	0.4854
		RF	0.2884	0.3447	0.2067	−0.0912
		SVM	0.1001	0.1207	0.0713	0.8663
AEC	Model_Ori	ANN	0.0087	0.0087	0.0045	
		RF	1.3584	1.3584	0.7112	
		SVM	0.0390	0.0390	0.0204	
	Model_Syn	ANN	0.0467	0.0467	0.0245	
		RF	0.1240	0.1240	0.0649	
		SVM	0.0497	0.0497	0.0260	
ALL	Model_Ori	ANN	0.6079	0.8605	0.3944	0.3615
		RF	0.6950	0.9025	0.5464	0.2976
		SVM	0.4793	0.6716	0.3073	0.6111
	Model_Syn	ANN	0.4504	0.6373	0.3147	0.6498
		RF	0.4779	0.6449	0.3427	0.6414
		SVM	0.4772	0.6673	0.3046	0.6160

**Table 4:** Coefficients for the corrosion growth model.

Para. (Variable)	Soil Category		
	C	CL	SCL
*t*_0_	3.05	3.06	2.57
*k*_0_	$5.51\times 10^{-1}$	$9.84\times 10^{-1}$	$5.99\times 10^{-1}$
*k*_1_ (*rp*)	$-8.98\times 10^{-5}$	$-1.06\times 10^{-4}$	$-1.82\times 10^{-4}$
*k*_2_ (*pH*)	$-5.90\times 10^{-2}$	$-1.15\times 10^{-1}$	$-6.42\times 10^{-2}$
*k*_3_ (*re*)	$-2.15\times 10^{-4}$	$-2.99\times 10^{-4}$	$-2.12\times 10^{-4}$
*k*_4_ (*cc*)	$8.38\times 10^{-4}$	$1.80\times 10^{-3}$	$8.62\times 10^{-4}$
*k*_5_ (*bc*)	$-1.28\times 10^{-3}$	$-4.88\times 10^{-4}$	$-6.78\times 10^{-4}$
*k*_6_ (*sc*)	$-5.33\times 10^{-5}$	$-2.09\times 10^{-4}$	$-1.13\times 10^{-4}$
*a*_0_	$8.85\times 10^{-1}$	$2.82\times 10^{-1}$	$9.65\times 10^{-1}$
*a*_1_ (*pp*)	$4.93\times 10^{-1}$	$4.61\times 10^{-1}$	$5.12\times 10^{-1}$
*a*_2_ (*wc*)	$3.72\times 10^{-3}$	$1.69\times 10^{-2}$	$4.50\times 10^{-4}$
*a*_3_ (*bd*)	$-1.01\times 10^{-1}$	$-9.87\times 10^{-2}$	$-1.58\times 10^{-1}$
*a*_4_ (*ct*)	$4.67\times 10^{-1}$	$5.67\times 10^{-1}$	$4.34\times 10^{-1}$

**Table 5:** Predictive performance of models built with different synthetic datasets.

		MAE	RMSE	MAPE	*R* ^2^
***D***_ctgan_	ANN	0.5884	0.8385	0.4203	0.3938
	RF	0.4753	0.6743	0.3429	0.6079
	SVM	0.5420	0.7299	0.3445	0.5405
***D***_em_	ANN	0.6182	0.8372	0.4219	0.3957
	RF	0.4763	0.6395	0.3609	0.6474
	SVM	0.5138	0.6801	0.3535	0.6011
***D***_ml_	ANN	0.4970	0.6801	0.3649	0.6011
	RF	0.4567	0.5937	0.3446	0.6668
	SVM	0.4756	0.6603	0.3129	0.6240

## Data Availability

All data that support the findings of this study are included in this manuscript.
